# Comprehensive LC-MS Analysis of the Most Abundant Flavonoid Glycosides in the Extracts of *Alcea rosea* Flowers

**DOI:** 10.3390/molecules31152737

**Published:** 2026-08-06

**Authors:** Weronika Wiśniewska, Monika Beszterda-Buszczak, Rafał Frański

**Affiliations:** 1 Faculty of Chemistry, Adam Mickiewicz University, Uniwersytetu Poznańskiego 8, 61-614 Poznań, Poland; 2 Department of Food Biochemistry and Analysis, Poznań University of Life Sciences, Mazowiecka 48, 60-623 Poznań, Poland; monika.beszterda@up.poznan.pl

**Keywords:** hollyhock, alcohol extracts, flavonoid glycosides, mass spectrometry, fragmentation pathways, liquid chromatography

## Abstract

The extracts of the *Alcea rosea* L. flowers are known to show various kinds of biomedical activities and are widely used as food and cosmetic ingredients. Unfortunately, no comprehensive analysis aimed at the identification of the most abundant flavonoid glycosides present in the extract of *Alcea rosea* L. has been made as of yet. In this paper we present the LC-MS identification of the most abundant flavonoid glycosides in the methanol and 75% ethanol extracts of *Alcea rosea* L. obtained from two manufacturers. The identification has been based on the detection of the respective parent and product ions as well as their relative abundances. The differences between identified isomeric and isobaric compounds have also been discussed. In all extracts a number of 3-*O*-anthocyanidin glycosides (anthocyanins) and 3-*O*-flavonol glycosides have been identified. Among the anthocyanins, the most abundant were malvidin glycosides, e.g., malvidin 3-*O*-(6″-[*E*]-*p*-coumaroyl)glucoside; among the flavonol glycosides, the most abundant were kaempferol glycosides, e.g., tiliroside.

## 1. Introduction

The species from the genus *Alcea* are ornamental garden plants grown for their beautiful, showy and very colorful flowers. Among these species, the *Alcea rosea* L., known as hollyhock, seems to be the most common; its flowers can be white, yellow, pink, red, purple and even almost black. Furthermore, the extracts of *Alcea rosea* L. have been widely studied with respect to their biomedical activities [1,2], and are widely used as food and cosmetic ingredients [3]. It is clear that the extract composition, and consequently its health-promoting effects, depend on the extraction conditions, mainly on the solvent used. The most common solvents used to prepare extracts of *Alcea rosea* L., further studied with respect to their biomedical properties, are methanol [4,5,6,7], methanol/water (~3/1) [8,9], ethanol/water (70–95% of ethanol) [10,11,12], and more rarely water [13,14]. Sometimes the dried alcohol extract has been subjected to further extraction with different solvents, e.g., with ethyl acetate [15].

The health-promoting properties of *Alcea rosea* L. extracts are strongly related to the high flavonoid content, e.g., anthocyanins (which are responsible for the beautiful dark color of the flowers), but also to other kinds of flavonoid glycosides. To the best of our knowledge, there is scarcely any comprehensive analysis aimed at the identification of the most abundant flavonoid glycosides present in the extract of *Alcea rosea* L., namely anthocyanins and other flavonoid glycosides. Anthocyanins have been identified in [5] and [14] (in the former in methanol extract, in the latter in acidified water extract), whereas the other flavonoid glycosides, or free aglycones, have been identified in [8,10,11] and [15]. As far as we know, there are only two reports in which both anthocyanins and other flavonoid glycosides have been identified [4,16]. For most of the identified compounds, only general names have been provided in [4] (e.g., anthocyanin I, anthocyanin II, kaempherol glycoside II, kaempherol glycoside III); thus, the information concerning the sugar moieties and their possible substitution sites has not been provided [4]. The only article in which both anthocyanins and other flavonoid glycosides have been carefully identified is that by Hosaka et al. [16]. The authors have identified nine anthocyanins, three flavonol glycosides and one flavone glycoside. In this paper we present the LC-MS identification of the most abundant flavonoid glycosides (anthocyanins and the others) in the extracts of *Alcea rosea* L. The identification has been based on the detection of the respective parent and product ions as well as their relative abundances. The differences between identified isomeric and isobaric compounds have also been discussed.

## 2. Results and Discussion

### 2.1. Identification of Anthocyanins

**Figure 1:**
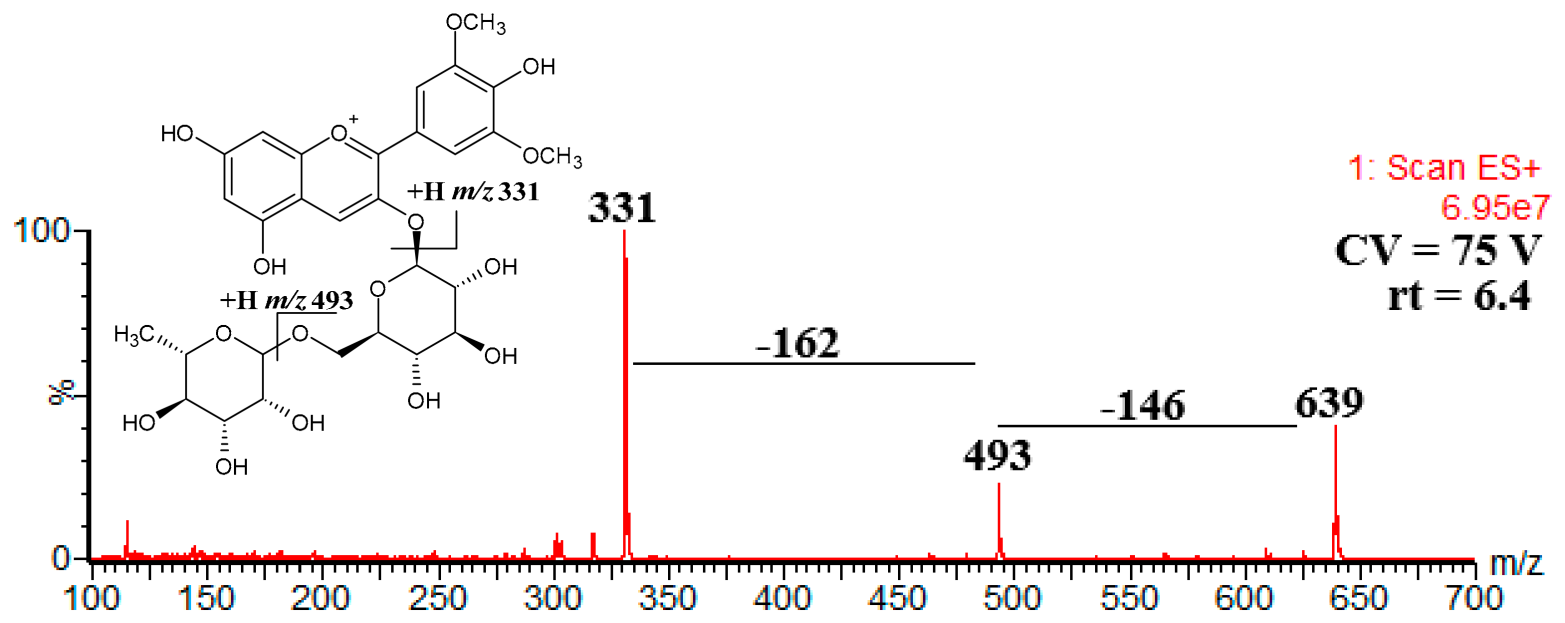
ESI(+) mass spectrum and observed fragmentation pattern of malvidin 3-*O*-rutinoside (**4**).

The first group of identified flavonoid glycosides were anthocyanins (anthocyanidin glycosides). Since they are flavylium cation-derived compounds, thus cationic compounds, their LC-MS analysis in the positive ion mode is more appropriate than in the negative ion mode. Four anthocyanidin 3-*O*-rutinosides (*O*-di-glycosides) were detected, as summarized in Table 1. These rutinosides have also been detected in other extracts of the hollyhock flowers [5,14,16]. The parent ions which were assigned as [M]^+^ (the assignment as [M−Cl]^+^ would also be acceptable [17]), yielded Y_1_^+^ and Y_0_^+^ product ions (the widely accepted nomenclature of glycoconjugate fragmentation [18]) with relative abundance, characteristic of 3-*O*-rutinosides [14,19,20]. The representative ESI(+) mass spectrum is shown in Figure 1.

**Figure 2:**
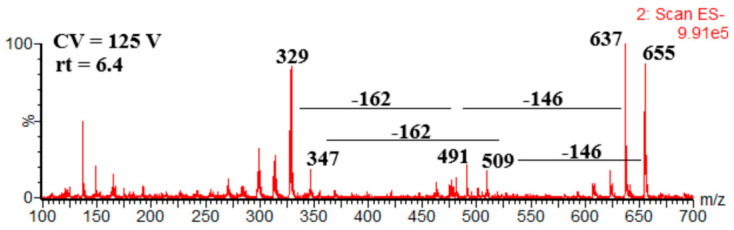
ESI(−) mass spectrum of malvidin 3-*O*-rutinoside (**4**). Peak at *m*/*z* 655 corresponds to the [M−2H+H_2_O]^−^ ion, and that at *m*/*z* 637 to the [M−2H]^−^ ion.

In the negative ion mode the anthocyanins were also detected; although, as expected, a relatively high background was observed since the sensitivity in negative ion mode was not as good as in positive ion mode. They yielded ions assigned as [M−2H+H_2_O]^−^ which enabled their differentiation from other flavonoid glycosides [21,22,23]. At higher cone voltage the ions [M−2H]^−^ were detected as well as the characteristic product ions, which fully confirmed the structures elucidated by the data obtained in the positive ion mode. The exemplary ESI(−) mass spectrum is shown in Figure 2.

### 2.2. Identification of Acylated Anthocyanins

Along with the anthocyanidin rutinosides **1**–**4**, four acylated anthocyanins were identified, namely petunidin 3-*O*-(6″-[*E*]-*p*-coumaroyl)glucoside (**5**), malvidin 3-*O*-(6″-[*E*]-*p*-coumaroyl)glucoside (**6**), petunidin 3-*O*-(6″-malonyl)glucoside (**7**), and malvidin 3-*O*-(6″-malonyl)glucoside (**8**) (Table 2).

The malonylated anthocyanin **8** has already been detected in the hollyhock extract [5,14,16], whereas the malonylated anthocyanin **7** has only been detected in [14]. To the best of our knowledge, the coumaroylated ones (**5** and **6**) have not been detected, although coumaric acid and its other conjugates have been detected in the hollyhock extract [4,10,11,15]. Compounds **5**–**8**, in contrast to the rutinosides **1**–**4**, did not yield Y_1_^+^ product ions; they yielded only Y_0_^+^ (Supplementary Material, Figure S1). Therefore, **5** and **6** can be easily differentiated from their isobaric compounds, **3** and **4**, respectively, by using low resolution mass spectrometry. Of course, the accurate masses (*m*/*z* values) obtained on the high-resolution instrument also enable their differentiation (Supplementary Material, Figure S2). In the negative ion mode, the acylated anthocyanins **5**–**8** (similarly to rutinosides **4**–**6**) yielded [M−2H+H_2_O]^−^ ions. Compounds **7** and **8**, in the negative ion mode, also yielded the product ions formed due to the decomposition of the malonyl moiety, namely [M−2H+H_2_O−CO_2_]^−^ and [M−2H−CO_2_]^−^ ions. In the Supplementary Material the single ion chromatograms are shown as representative examples (Figures S3 and S4).

### 2.3. Identification of Flavonol Glycosides

**Figure 3:**
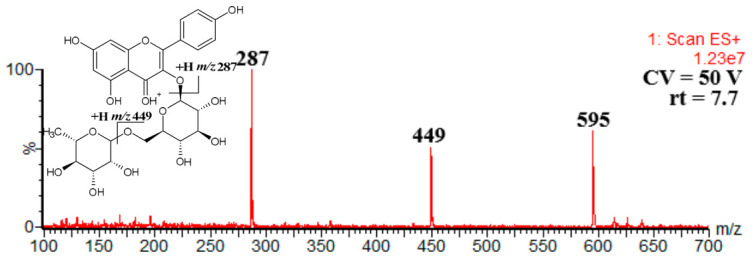
The ESI(+) mass spectrum of kaempferol 3-*O*-rutinoside (**7**).

In the analyzed extracts, the glycosides of three flavonols, namely glucosides and rutinosides of kaempferol, quercetin and myricetin, were detected (Table 3). Hosaka et al. have detected myricetin 3-*O*-glucoside and kaempferol 3-*O*-glucoside/rutinoside [16]. The flavonol glycosides yielded intense signals in both positive and negative ion mode ([M+H]^+^ and [M−H]^−^ ions, respectively). The ESI(+) mass spectrum of kaempferol 3-*O*-rutinoside (**9**) is shown in Figure 3 as a representative example.

The fragmentation pathways of flavonol 3-*O*-rutinosides observed in the positive ion mode resemble those reported elsewhere [19,24,25,26]. In the negative ion mode, they yielded abundant [Y_0_−H]^−•^ ion, characteristic of flavonol 3-*O*-glycosides (Figure S5) [27,28,29]. The negative ions enable their differentiation from their isomeric anthocyanins (**1** and **9**, **2** and **11**, **3** and **13**, Table 1 and Table 3). It has to be stressed that the identification of quercetin 3-*O*-rutinoside (**11**, rutin) and quercetin 3-*O*-glucoside (**12**, isoquercetin) was also confirmed by comparison of their retention times and mass spectra with those of the standards. Furthermore, myricetin 3-*O*-glucoside (**14**) had a slightly different retention time than myricetin 3-*O*-galactoside identified in the extract of *Trifolium repens* (galactose is the second most common hexose after glucose which is substituted onto the phenolic compounds in plants) [28]. Therefore, in the detected compounds the hexose has always been tentatively identified as glucose.

**Figure 4:**
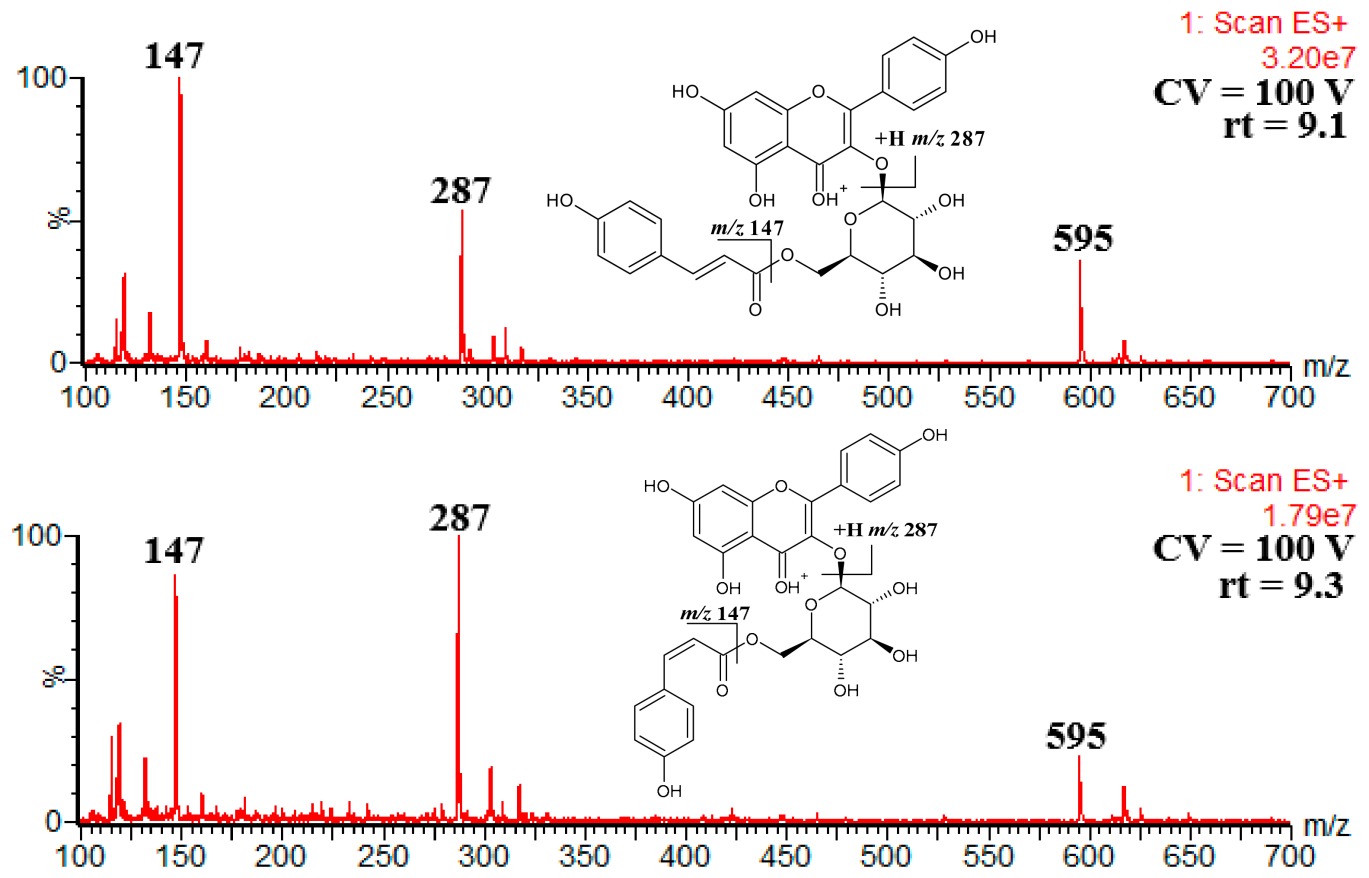
The ESI(+) mass spectra of isomers **15a** and **15b** (rt = 9.1 and rt = 9.3, respectively) obtained in the positive ion mode.

Besides the flavonol glycosides **9**–**14**, two coumaroylated conjugates of kaempferol glucoside were identified, namely kaempferol 3-*O*-(6″-[*E*]-*p*-coumaroyl)glucoside (*trans*-tiliroside, **15a**) and kaempferol 3-*O*-(6″-[*Z*]-*p*-coumaroyl)glucoside (*cis*-tiliroside, **15b**). The isomer *trans* has already been detected by Abdel-Salam et al. [10]. Compounds **15a**/**b** are isobaric to compound **9** and to the M^+^ ion of **1**. The single ion chromatograms of these four compounds as well as their high-resolution mass spectra are shown in the Supplementary Material (Figures S6 and S7, respectively). It is known that under the reversed-phase chromatography conditions, the *trans*-tiliroside (**15a**) is eluted earlier than *cis*-tiliroside (**15b**) [30,31,32]. The ESI mass spectra of isomers **15a** and **15b** were different with respect to the relative ion abundances, especially in the positive ion mode, as shown in Figure 4 (in the negative ion mode the differences were less pronounced, Figure S8). Isomer *trans* yielded a much more abundant product ion at *m*/*z* 147 ([HO-C_6_H_4_-CH=CH-CO]^+^) in comparison to the product ion at *m*/*z* 287 (Y_0_^+^ ion) than isomer *cis*. Therefore, these isomers can be differentiated on the basis of relative ion abundances.

### 2.4. Identification of Other Flavonoid Glycosides

**Figure 5:**
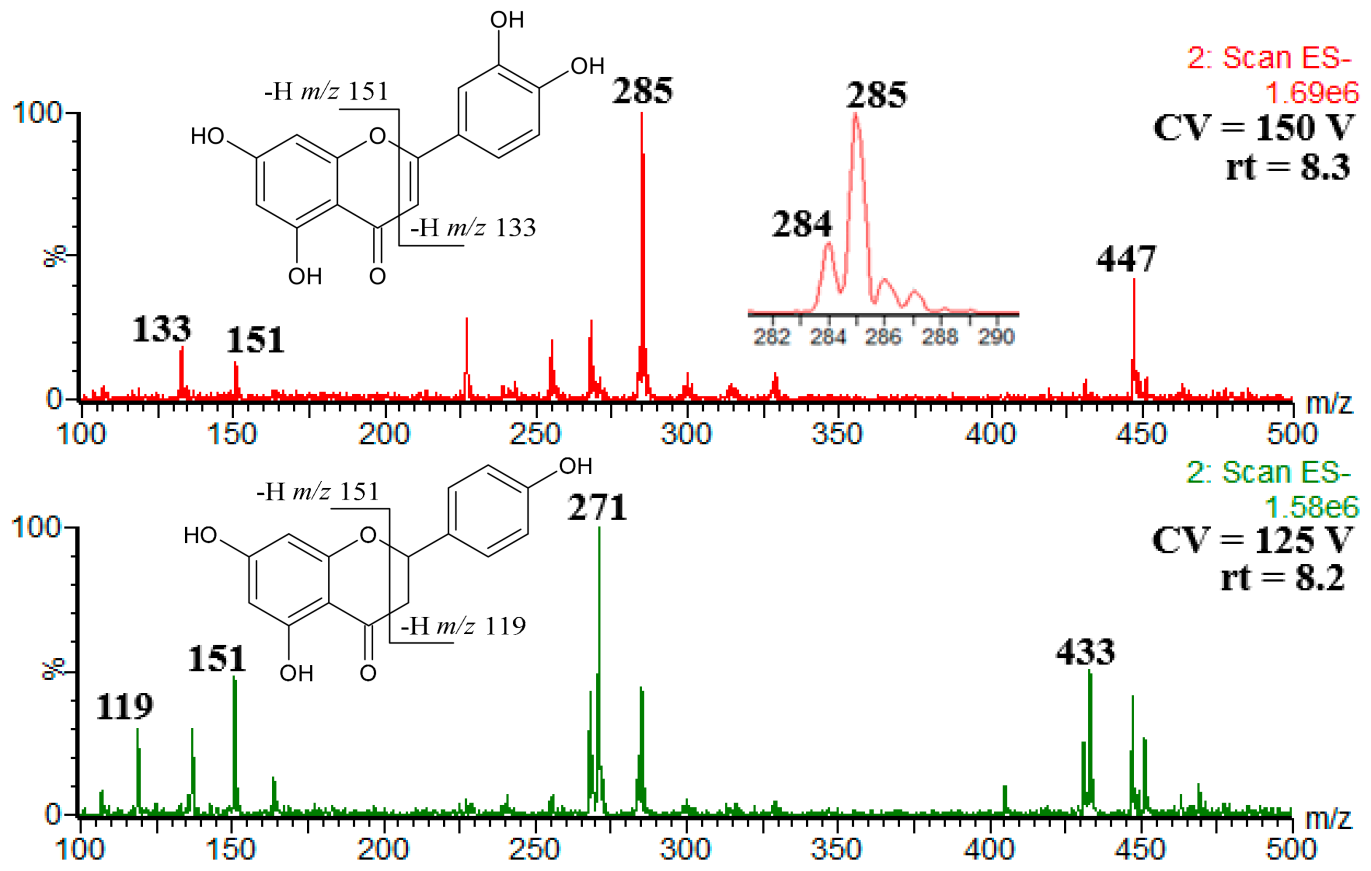
ESI(−) mass spectra of **17** and **18** and their aglycone fragmentation patterns.

Apart from anthocyanins (**1**–**8**) and flavonol glycosides (**9**–**15**), three other flavonoid glycosides were identified: two flavone glycosides, namely apigenin 7-*O*-glucoside (**16**) and luteolin 4′-*O*-glucoside (**17**), and one flavanone glycoside, namely naringenin 7-*O*-glucoside (**18**) (Table 4). Of these three compounds, Hosaka et al. have detected luteolin 4′-*O*-glucoside [16]. Although these compounds had very similar retention times (Supplementary Material, Figure S9) it was possible to determine their structures, as the retention times of parent and product ions were carefully checked and they always matched perfectly. In the negative ion mode apigenin 7-*O*-glucoside (**16**) yielded the abundant [Y_0_−H]^−•^ product ion (*m*/*z* 268, Table 3); however, its relative abundance was lower than that of flavonol 3-*O*-glycosides (Table 2). Low abundant [Y_0_−H]^−•^ ion (*m*/*z* 284), in comparison to the Y_0_^−^ ion (*m*/*z* 285), was detected for **17,** indicating the glycosylation at the C-4′ site [27,33,34]. The lack of the [Y_0_−H]^−•^ ion and the *m*/*z* values of Y_0_^+^/Y_0_^−^ ions (275/273) indicated the flavanone aglycone for **17** [19,34,35]. At high cone voltage, the fragmentation of Y_0_^−^ ions of **17** and **18** (these ions correspond to the deprotonated aglycones) enabled the determination of aglycone structures as shown in Figure 5. The product ion at *m*/*z* 133 detected for **17** confirmed luteolin as the aglycone, and excluded its isomer kaempferol [36,37], and the product ions at *m*/*z* 119 and 151 confirmed naringenin as the aglycone for **18** [19,38]. Naringenin 7-*O*-glucoside (**18**), commonly known as prunin (a compound with very promising anti-cancer activity, [39]), is present in high amounts in citrus fruits and in plants belonging to the Prunus genus [40]. We have confirmed its presence in the hollyhock extracts by comparing its retention time with that present in the bark extract of *Prunus persica* [19].

### 2.5. Relative Abundances of the Identified Flavonoid Glycosides

Two samples of *Alcea rosea* L. (obtained from two different manufacturers) were extracted with methanol (samples **1A** and **2A**) and with 75% ethanol (samples **1B** and **2B**). These are the most common solvents used for the preparation of *Alcea rosea* L extracts. In order to semi-quantitively evaluate the relative abundances of the identified flavonoid glycosides in the analyzed samples, the chromatographic peak areas of [M]^+^ ions for anthocyanins, and [M+H]^+^ as well as [M−H]^−^ ions for other flavonoid glycosides, obtained at a low cone voltage (40 V) in order to avoid fragmentation, were compared. Since anthocyanins yields low signals in the negative ion mode (ions [M−2H+H_2_O]^−^), in this mode they were not taken into account.

**Figure 6:**
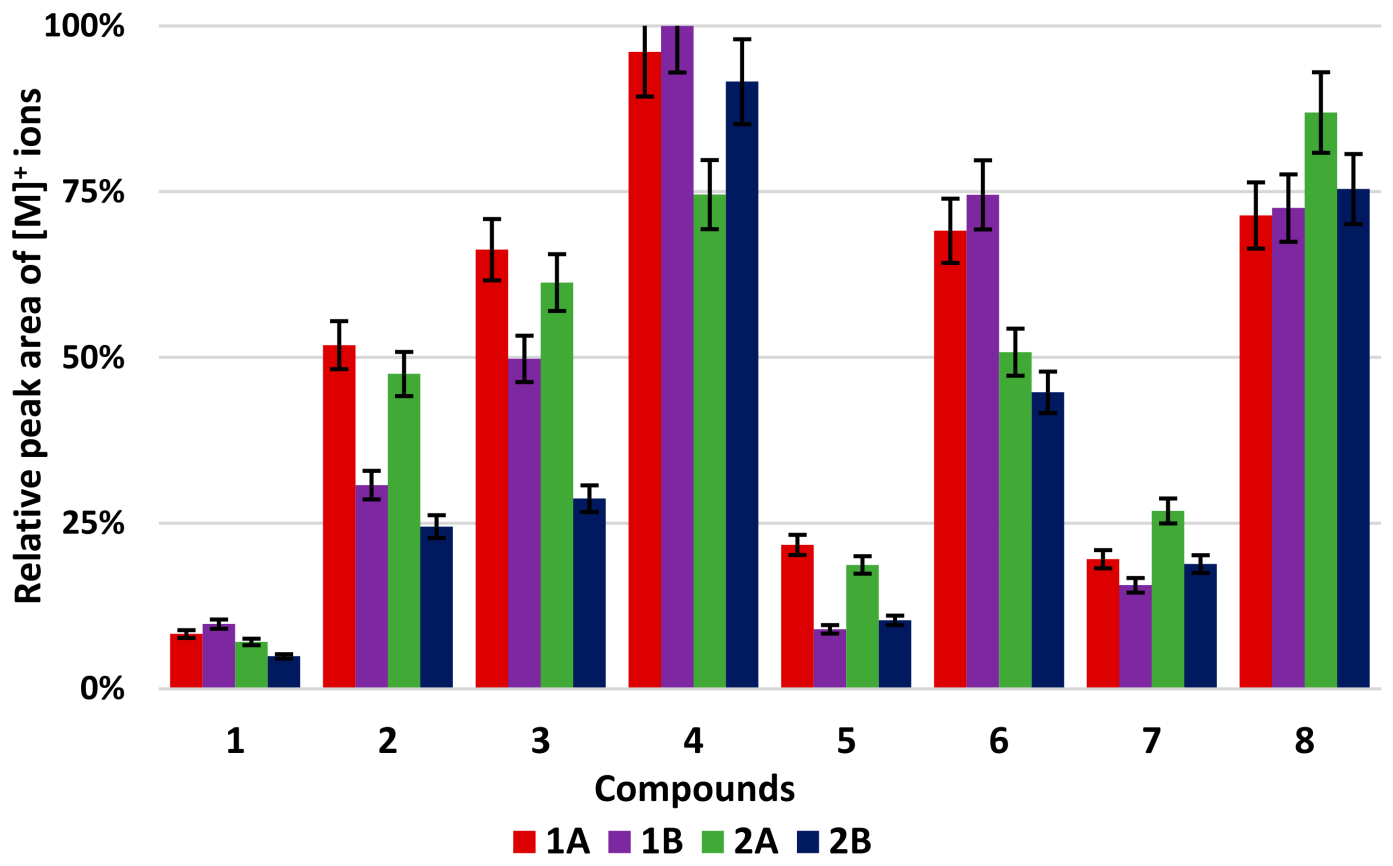
Comparison of the chromatographic peak areas of [M]^+^ ions of identified anthocyanins.

Figure 6 shows the chromatographic peak areas obtained for anthocyanins (compounds **1**–**8**). It is reasonable that their ESI responses are comparable, since it is the group of compounds of the same type (flavylium cation-containing compounds). In all analyzed extracts the most abundant are malvidin 3-*O*-rutinoside (**4**) and malvidin 3-*O*-(6″-malonyl)glucoside (**8**). The relative amount of malvidin 3-*O*-(6″-[*E*]-*p*-coumaroyl)glucoside (**6**) is also quite high, although in the extracts **2A** and **2B** (manufacturer 2) their amounts are smaller than in the extracts **1A** and **1B** (manufacturer 1). Therefore, hollyhock can be regarded as an excellent source of malvidin glycosides, compounds of well-documented health-promoting properties, e.g., anti-cancer and anti-inflammatory [41,42]. The effect of the solvent has not been pronounced for malvidin glycosides, e.g., the relative amount of compound **4** was the lowest in sample **2A** (manufacturer 2, methanol extract), whereas for compound **8** we deal with the opposite situation (Figure 6). On the other hand, the effect of solvent has been clearly pronounced for **2**, **3**, **5** and **7**, namely, in the methanol extracts (samples **1A** and **2A**) their relative amounts are higher than in the 75% ethanol (samples **1B** and **2B**, Figure 6).

**Figure 7:**
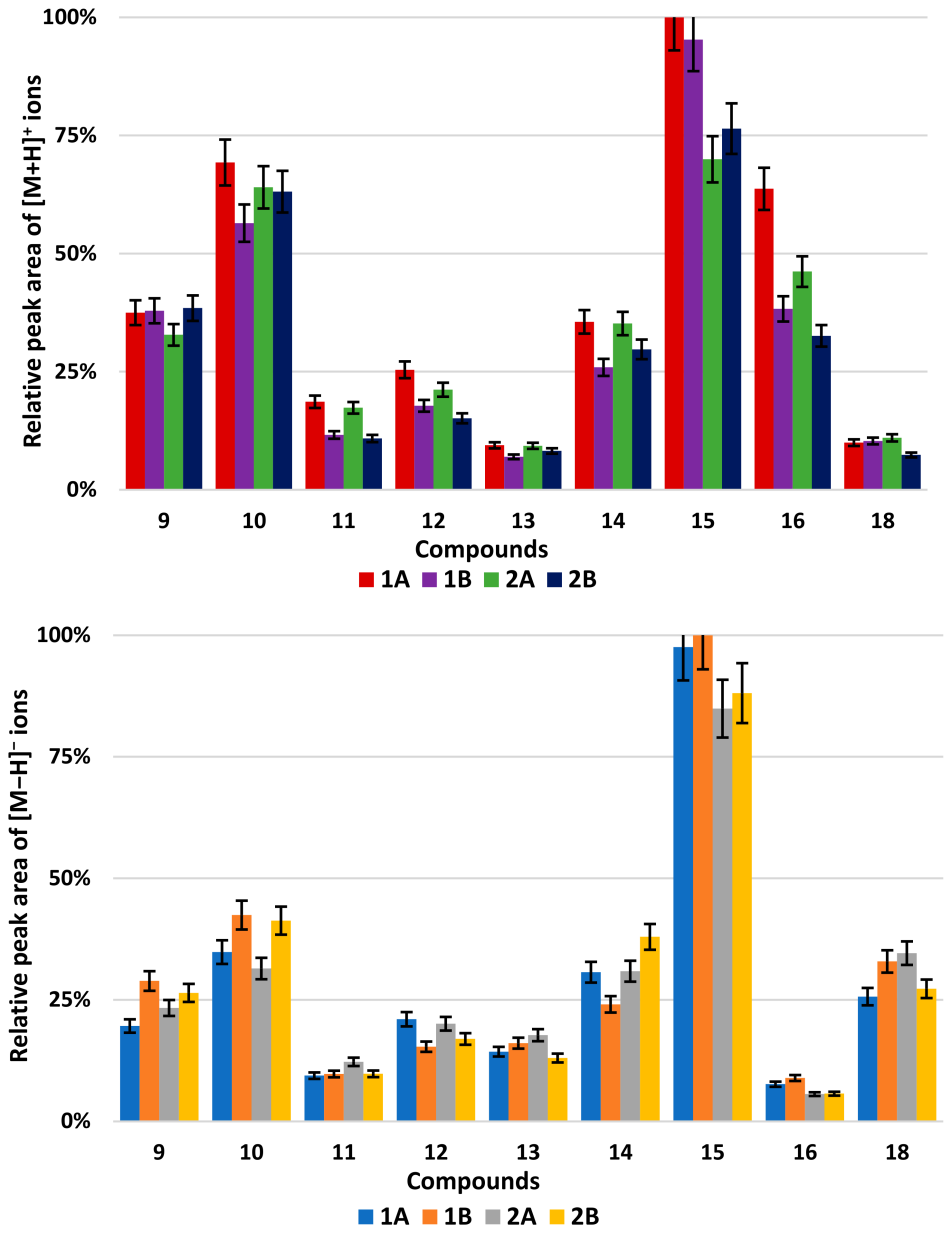
Comparison of the chromatographic peak areas of [M+H]^+^ and [M−H]^−^ ions of identified flavonoid glycosides.

Figure 7 shows the chromatographic peak areas of [M+H]^+^ and [M−H]^−^ ions of compounds **1**–**16** and **18**. Compound **17** is of low abundance and has been poorly separated from its isomer **10** (Figure S7); therefore, it was not included. Compounds **15a** and **15b** (*trans*/*cis*-tiliroside, respectively), which also have been poorly separated (Figure S5), have been combined as one compound **15**. The results obtained in both positive and negative ion mode indicate that among compounds **1**–**18**, the most abundant are kaempferol glycosides (**9**, **10**, **15**), and among them the most abundant is tiliroside **15**, a compound of great potential to mitigate inflammation and its associated diseases [43,44]. The effect of the solvent has not been clearly pronounced for flavonoid glycosides **1**–**18**. In the positive ion mode, usually in the methanol extract, the peak areas are slightly higher; however, in the negative ion mode it has not been observed.

**Scheme 1:**
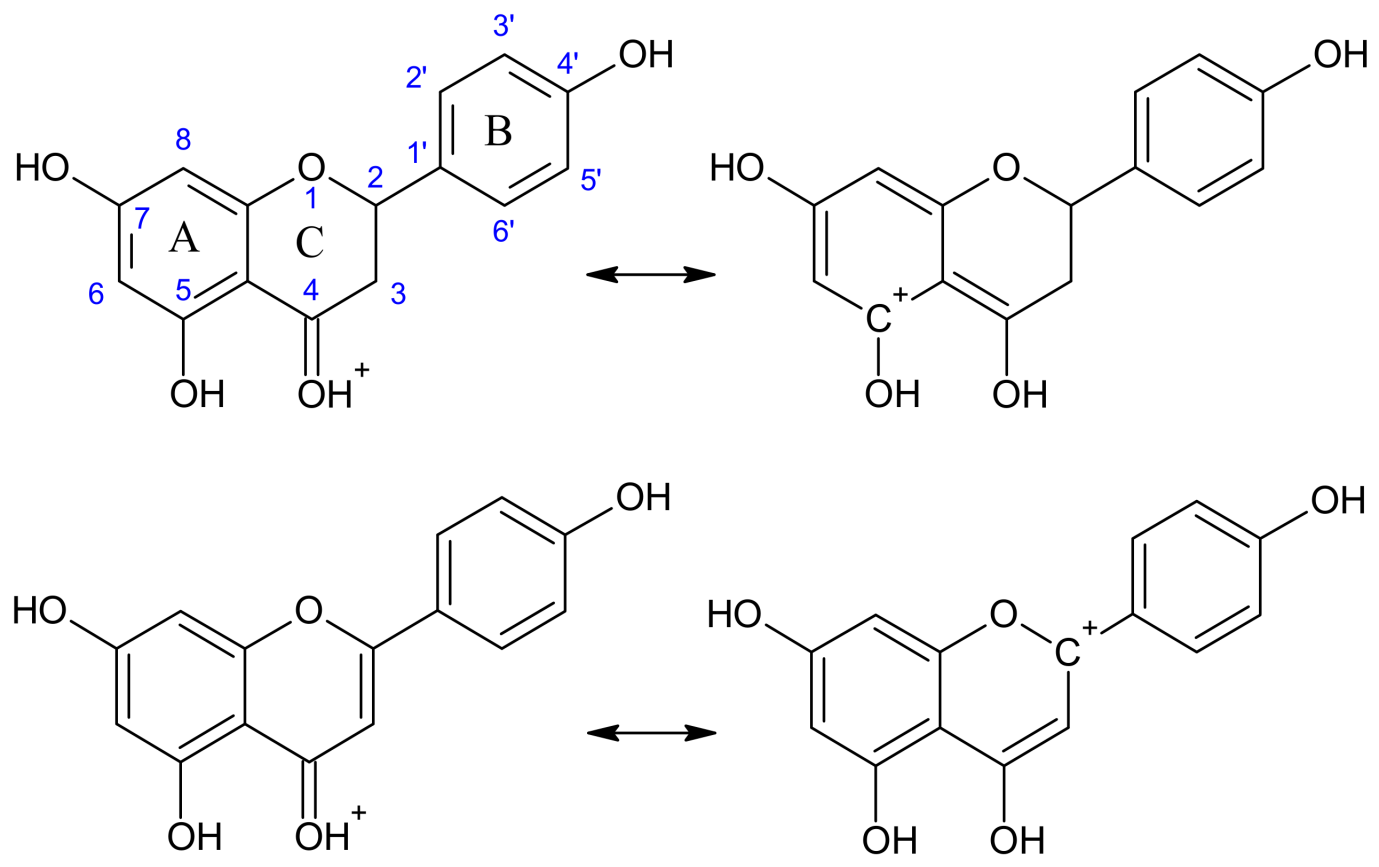
Examples of the possible resonance structures of protonated naringenin (**top**) and protonated apigenin (**bottom**).

Compounds **9**–**15** are flavonol 3-*O*-glycosides so it is reasonable that they have very similar ESI response in both positive and negative ion mode. It is known that the C4=O moiety (carbonyl oxygen atom) is the most favored protonation site of flavonoid molecules [45,46], which for **9**–**15** can be affected by the interaction with neighboring groups C5-OH and glycosylated C3-OH. Deprotonation of **9**–**15** can occur at phenolic groups substituted either at ring A or B [28,47]. In both ESI(+) and ESI(−), the relative abundances of **9**–**15** were similar (Figure 7). On the other hand, for compounds **16** and **18** there is a notable discrepancy between their relative amounts observed in the positive and negative ion mode (Figure 7). Compound **16** (apigenin 7-*O*-glucoside) contains only two free phenolic groups (C5-OH and C4′-OH); therefore, it is reasonable that its ESI(−) response is lower than that of **9**–**15** (consequently the ESI(+) response can be higher). Compound **18** (naringenin 7-O-glucoside), among the identified compounds, is the only one which contains a single C2-C3 bond at the C ring (flavanone). Therefore, the protonation of its C4=O moiety cannot yield a structure containing the charge delocalized in the B ring (in contrast to the flavones and flavonols, Scheme 1); thus, compound **18** showed a lower ESI(+) response than **1**–**17**, and as a consequence, a higher ESI(−) response. Of course, protonated **18** and the others can yield structures containing the charge delocalized in ring A (Scheme 1).

## 3. Materials and Methods

### 3.1. Preparation of the Extracts for HPLC-MS Analysis

Two dietary supplements, dried black mallow flowers (*Malvae arboreae cum calycibus flos*), sourced from various manufacturers, were purchased from local pharmacies in the first quarter of 2026. A 0.5 g portion of each sample was extracted with 15 mL of 75% ethanol. The sample was then homogenized (T 18 Ultra Turrax, IKA-Werke, Staufen, Germany), shaken at 500 rpm for 30 min (Vortex 3, IKA-Werke GmbH, Staufen, Germany) and after 24 h filtered through a 0.45 μm syringe filter (Macherey-Nagel GmbH, Düren, Germany). The same procedure was followed for the extraction using pure methanol. All extracts were stored at a temperature of 5 °C.

### 3.2. HPLC-MS Analysis

The HPLC-MS analyses were performed using a Waters Arc HPLC pump and a Waters SQD mass spectrometer (single quadrupole-type instrument equipped with electrospray ionization (ESI) source, Z-spray, Milford, MA, USA). The software used was MassLynx V4.2 SCN1046 (Milford, MA, USA). Using an autosampler, the sample solutions were injected into the XTerra^®^ MS C18 column (Waters, Milford, MA, USA, 5 μm, 150 mm × 3 mm i.d.). The injection volume was 10 µL. The solutions were analyzed by using a linear gradient of CH_3_CN-H_2_O at a flow rate of 0.5 mL/min. The gradient started from 0% CH_3_CN to 95% H_2_O with 5% of a 10% solution of formic acid in water, reaching 95% CH_3_CN after 15 min, and the latter concentration was maintained for 10 min. The ESI mass spectra were recorded in the *m*/*z* range 100–1000, in positive and negative modes simultaneously (during the HPLC/ESI-MS analyses, the mass spectrometer was switched in the fast mode between the positive and negative ion modes). The ESI source potentials were as follows: capillary, 3 kV; lens, 0.5 V; extractor, 4 V; cone voltage (CV) 30–150 V. The latter parameter has the greatest impact on the full scan mass spectra recorded. An increase in this parameter leads to the so-called “in-source” fragmentation/dissociation, but a low cone voltage that is too low may cause a decrease in sensitivity. The source temperature was 120 °C, and the desolvation temperature was 300 °C. Nitrogen was used as the nebulizing and desolvating gas at flow rates of 100 and 300 L h^−1^, respectively. In order to evaluate the relative abundances of identified compounds, the HPLC-MS analyses were performed in triplicate, and the relative standard deviation did not exceed 7%.

### 3.3. HPLC-QTOF-MS Analysis

In order to confirm the elemental composition of the identified compounds the HPLC-QTOF-MS analyses were performed using an UltiMateTM 3000 UHPLC system (ThermoScientific/Dionex, Sunnyvale, CA, USA) and an Impact HD mass spectrometer (QTOF type instrument equipped with electrospray ion source; Bruker Daltonics, Billerica, MA, USA). Using an autosampler, the sample solutions were injected into the Kinetex C18 column (100 × 2.10 mm i.d., 2.6 μm particle size). The mobile phases used were water with 0.1% formic acid (solvent A) and acetonitrile with 0.1% formic acid (solvent B). The samples were analyzed using a linear gradient at a flow rate of 0.3 mL/min and the column temperature was maintained at 35 °C. The gradient was programmed as follows: 90% A and 10% B for 1 min, followed by a linear change to 5% A and 95% B in 20 min, held for 10 min. The instrument was operated in the positive ion mode, under the following optimized settings: end-plate voltage 500 V, capillary voltage 4.2 kV; collision energy 8 eV, nebulizer pressure 1.5 bar; dry gas (nitrogen); temperature 200 °C; dry gas flow rate 8 L/min.

## 4. Conclusions

Anthocyanidin 3-*O*-glycosides, mainly malvidin glycosides, and flavonol 3-*O*-glycosides, mainly kaempferol glycosides, have been found as main flavonoid conjugates in the extracts of the flowers of *Alcea rosea* L. No significant differences between the methanol and 75% ethanol extract compositions have been observed. Among the identified compounds, the acylated conjugates were quite abundant, namely malvidin 3-*O*-(6″-malonyl)glucoside, malvidin 3-*O*-(6″-[*E*]-*p*-coumaroyl)glucoside and kaempferol 3-*O*-(6″-*p*-coumaroyl)glucoside (*trans*- and *cis*-tiliroside). Therefore, the flowers of the *Alcea rosea* L. seem to be a good source of malvidin and kaempferol conjugates. It has been demonstrated that the detected isomers, e.g., delphinidin 3-*O*-rutinoside and quercetin-*O*-rutinoside (rutin), as well as isobars, e.g., kaempferol-*O*-rutinoside and tiliroside, can be differentiated on the basis of low-resolution mass spectra by using the cone-voltage-induced fragmentation.

## Figures and Tables

**Table 1:** HPLC-ESI-MS(+) results obtained for anthocyanidin 3-*O*-rutinoside.

	Compound (Retention Time, min)	Ion *m*/*z*, % ri (CV = 75 V)
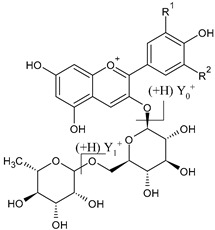	R^1^ = OH, R^2^ = H Cyanidin 3-*O*-rutinoside (**1**) (6.0 min)	[M]^+^ 595, 25%; Y_1_^+^ 449, 25%; Y_0_^+^ 287, 100%
	R^1^ = OH, R^2^ = OH Delphinidin 3-*O*-rutinoside (**2**) (5.8 min)	[M]^+^ 611, 35%; Y_1_^+^ 465, 25%; Y_0_^+^ 303, 100%
	R^1^ = OCH_3_, R^2^ = OH Petunidin 3-*O*-rutinoside (**3**) (6.1 min)	[M]^+^ 625, 40%; Y_1_^+^ 479, 25%; Y_0_^+^ 317, 100%
	R^1^ = OCH_3_, R^2^ = OCH_3_ Malvidin 3-*O*-rutinoside (**4**) (6.4 min)	[M]^+^ 639, 40%; Y_1_^+^ 493, 25%; Y_0_^+^ 331, 100%

**Table 2:** HPLC-ESI-MS(+) results obtained for acylated anthocyanins.

Compound (Retention Time, min)	Ion *m*/*z*, % ri (CV = 100 V)
Petunidin 3-*O*-(6″-[*E*]-*p*-coumaroyl)glucoside (**5**) (7.6)	[M]^+^ 625, 45%; Y_0_^+^ 317, 100%
Malvidin 3-*O*-(6″-[*E*]-*p*-coumaroyl)glucoside (**6**) (7.9)	[M]^+^ 639, 30%; Y_0_^+^ 331, 100%
Petunidin 3-*O*-(6″-malonyl)glucoside (**7**) (6.7)	[M]^+^ 565, 25%; Y_0_^+^ 317, 100%
Malvidin 3-*O*-(6″-malonyl)glucoside (**8**) (6.9)	[M]^+^ 579, 50%; Y_0_^+^ 331, 100%

**Table 3:** HPLC-ESI-MS(−/+) results obtained for flavonol 3-*O*-glycosides (CV = 75 V).

Compound (Retention Time, min)	(+) Ion *m*/*z*, % ri CV= 50 V	(−) Ion m/z, % ri CV= 125 V
Kaempferol 3-*O*-rutinoside (**9**) (7.7 min)	[M+H]^+^ 595, 70%; Y_1_^+^ 449, 50%; Y_0_^+^ 287, 100%	[M−H]^−^ 593, 90%; Y_0_^−^ 285, 50%; [Y_0_−H]^−•^ 284, 100%
Kaempferol 3-O-glucoside (**10**) (8.0 min)	[M+H]^+^ 449, 50%; Y_0_^+^ 287, 100%	[M−H]^−^ 447, 100%; Y_0_^−^ 285, 40%; [Y_0_−H]^−^ 284, 80%
Quercetin 3-*O*-rutinoside (rutin, **11**) (7.3 min)	[M+H]^+^ 611, 80%; Y_1_^+^ 465, 60%; Y_0_^+^ 303, 100%	[M−H]^−^ 609, 90%; Y_0_^−^ 301, 40%; [Y_0_−H]^−•^ 300, 100%
Quercetin 3-*O*-glucoside (isoquercetin, **12**) (7.6 min)	[M+H]^+^ 465, 50%; Y_0_^+^ 303, 100%	[M−H]^−^ 463, 80%; Y_0_^−^ 301, 40%; [Y_0_−H]^−•^ 300, 100%
Myricetin 3-*O*-rutinoside (**13**) (6.9 min)	[M+H]^+^ 627, 75%; Y_1_^+^ 481, 55%; Y_0_^+^ 319, 100%	[M−H]^−^ 625, 100%; Y_0_^−^ 317, 30%; [Y_0_−H]^−•^ 316, 100%
Myricetin 3-*O*-glucoside (**14**) (7.1 min)	[M+H]^+^ 481, 60%; Y_0_^+^ 319, 100%	[M−H]^−^ 479, 100%; Y_0_^−^ 317, 30%; [Y_0_−H]^−•^ 316, 75%

**Table 4:** HPLC-ESI-MS(−/+) results obtained for other flavonoid glycosides.

Compound (Retention Time, min)	(+) Ion *m*/*z*, % ri	(−) Ion *m*/*z*, % ri
Apigenin 7-*O*-glucoside (**16**) (8.1 min)	[M+H]^+^ 433, 35%; Y_0_^+^ 271, 100% (CV = 75 V)	[M−H]^−^ 431, 100%; Y_0_^−^ 269, 60%; [Y_0_−H]^−•^ 268, 60% (CV = 100 V)
Luteolin 4′-*O*-glucoside (**17**) (8.3 min)	[M+H]^+^ 449, 95%; Y_0_^+^ 287, 100% (CV = 50 V)	[M−H]^−^ 447, 40%; Y_0_^−^ 285, 100%; [Y_0_−H]^−•^ 284, 25% (CV = 150 V)
Naringenin 7-*O*-glucoside (**18**) (8.2 min)	[M+H]^+^ 435, 40%; Y_0_^+^ 273, 100% (CV= 50 V)	[M−H]^−^ 433, 50%; Y_0_^−^ 271, 100% (CV = 125 V)

## Data Availability

The original contributions presented in this study are included in the article and Supplementary Materials. Further inquiries can be directed to the corresponding author (R.F.).

## Supplementary Materials

The following supporting information can be downloaded at: https://www.mdpi.com/article/10.3390/molecules31152737/s1.
