# Supplementary material for: Comprehensive LC-MS Analysis of the Most Abundant Flavonoid Glycosides in the Extracts of Alcea rosea Flowers
Source: Molecules. 2026 Aug 6;31(15):2737. doi: 10.3390/molecules31152737 (PMC13467696; doi:10.3390/molecules31152737)
Supplement: Supplementary file 1 [file molecules-31-02737-s001.zip › molecules-4467476-supplementary.pdf]

Comprehensive LC-MS analysis of flavonoid glycosides in the extracts of *Alcea rosea* flowers

SUPPLEMENTARY MATERIAL

Weronika Wiśniewska, Monika Beszterda-Buszczak, Rafał Frański

Table of contents:

Figure S1. ESI mass spectra of 3-*O*-(6″-malonyl)glucoside (**5**) and malvidin 3-*O*-(6″-[*E*]-*p*-coumaroyl)glucoside (**6**)……………………………………………………………………..**2**

Figure S2. The high resolution mass spectra of **4** and **6** (the accurate *m/z* values of $[M]^+$ ions)...**2**

Figure S3. Single ion chromatograms obtained for malvidin 3-*O*-rutinoside (**4**, rt = 6.4 min) and malvidin 3-*O*-(6″-[E]-*p*-coumaroyl)glucoside (**6**, rt = 7.9 min), $[M{-}2H{+}H_2O]^-$ *m/z* 655, $[M]^+$ *m/z* 639, Y_1^+ *m/z* 493, Y_0^+ *m/z* 331……………………………………………………………………………………...**3**

Figure S4. Exemplary single ion chromatograms obtained for malvidin 3-*O*-(6″-malonyl)glucoside (**7**), $[M]^+$ *m/z* 579, $[M{-}2H{+}H_2O{-}CO_2]^-$ *m/z* 551, $[M{-}2H{-}CO_2]^-$ *m/z* 533...**4**

Figure S5. The ESI(−) mass spectrum of kaempferol 3-*O*-rutinoside (**7**)………………………**4**

Figure S6. Single ion chromatograms obtained in the positive (*m/z* 595) and negative ion mode (*m/z* 593) for cyanidin 3-*O*-rutinoside (**1**, 6.0 min), kaempferol 3-*O*-rutinoside (**7**, 7.7 min), kaempferol 3-*O*-(6″-[*E*]-*p*-coumaroyl)glucoside (isomer *trans*, **15a**, 9.1 min) and kaempferol 3-*O*-(6″-[*Z*]-*p*-coumaroyl)glucoside (isomer *cis*, **15b**, 9.3 min)……………………….………**5**

Figure S7. The high resolution mass spectra of **1**, **9**, **15a** and **15b** ……………………………..……………………………………………..……………….**6**

Figure S8. The ESI(−) mass spectra of isomers **15a** and **15b** (rt = 9.1 and rt = 9.3, respectively) obtained in the negative ion mode……………………………………………………………...**6**

Figure S9. Single ion chromatograms obtained for $[M{-}H]^-$ ions of apigenin 7-*O*-glucoside (**16**, *m/z* 431, rt = 8.1 min), luteolin 4'-*O*-glucoside (**17**, *m/z* 447, rt = 8.3 min), kaempferol 3-*O*-glucoside (**10**, *m/z* 447, rt = 8.0 min), and naringenin 7-*O*-glucoside (**18**, *m/z* 433, rt = 8.2 min)…………………………………………………………………………………….....**7**

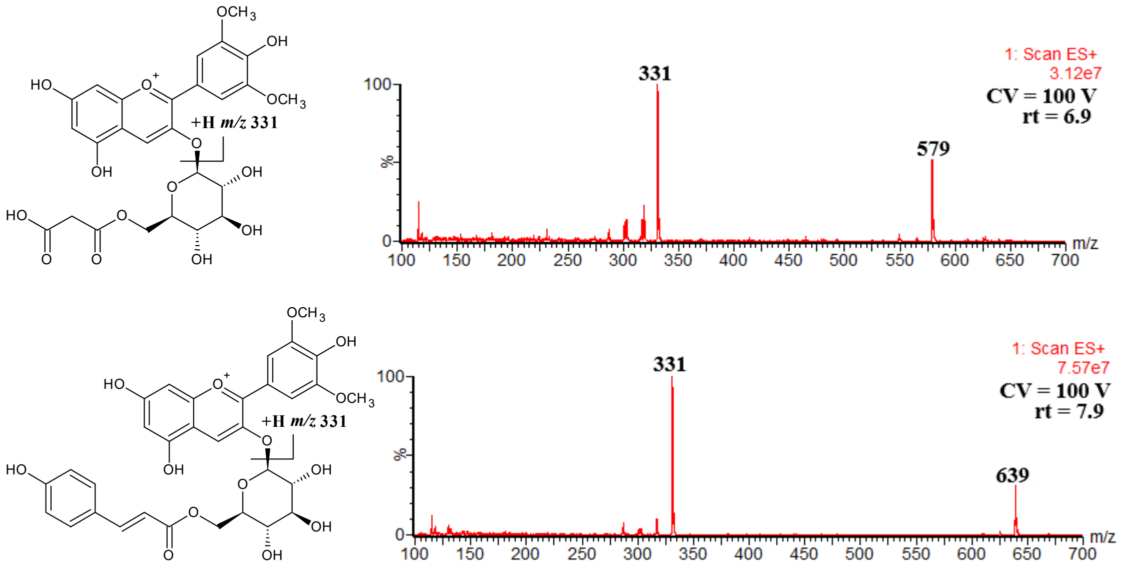

Figure S1. ESI mass spectra of 3-O-(6″-malonyl)glucoside (**5**) and malvidin 3-O-(6″-[*E*]-*p*-coumaroyl)glucoside (**6**).

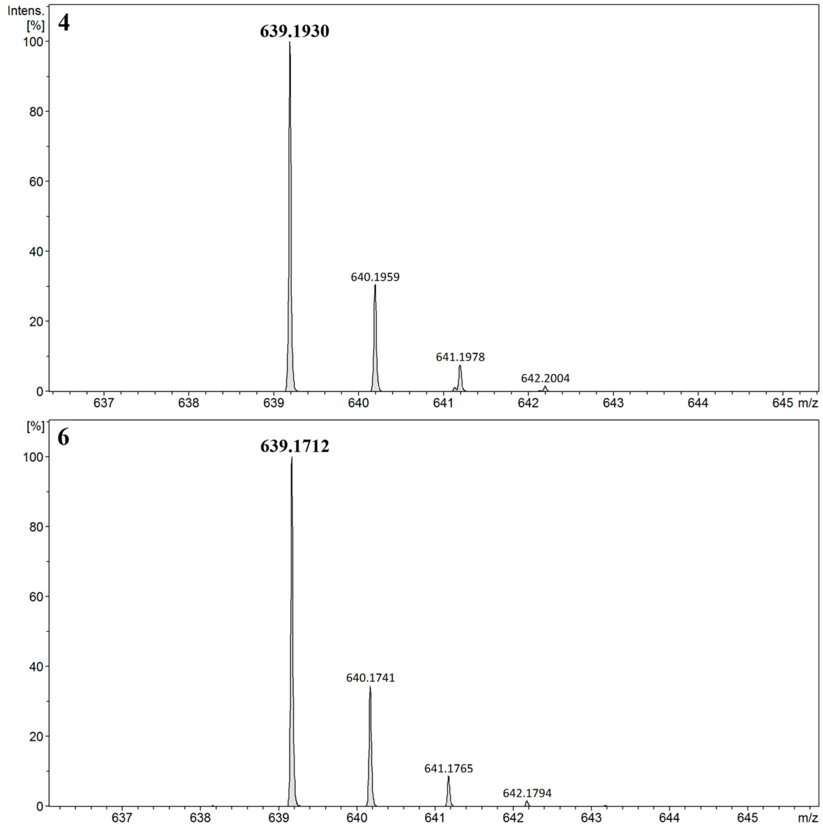

Figure S2. The high resolution mass spectra of **4** and **6** (the accurate *m/z* values of $[M]^+$ ions).

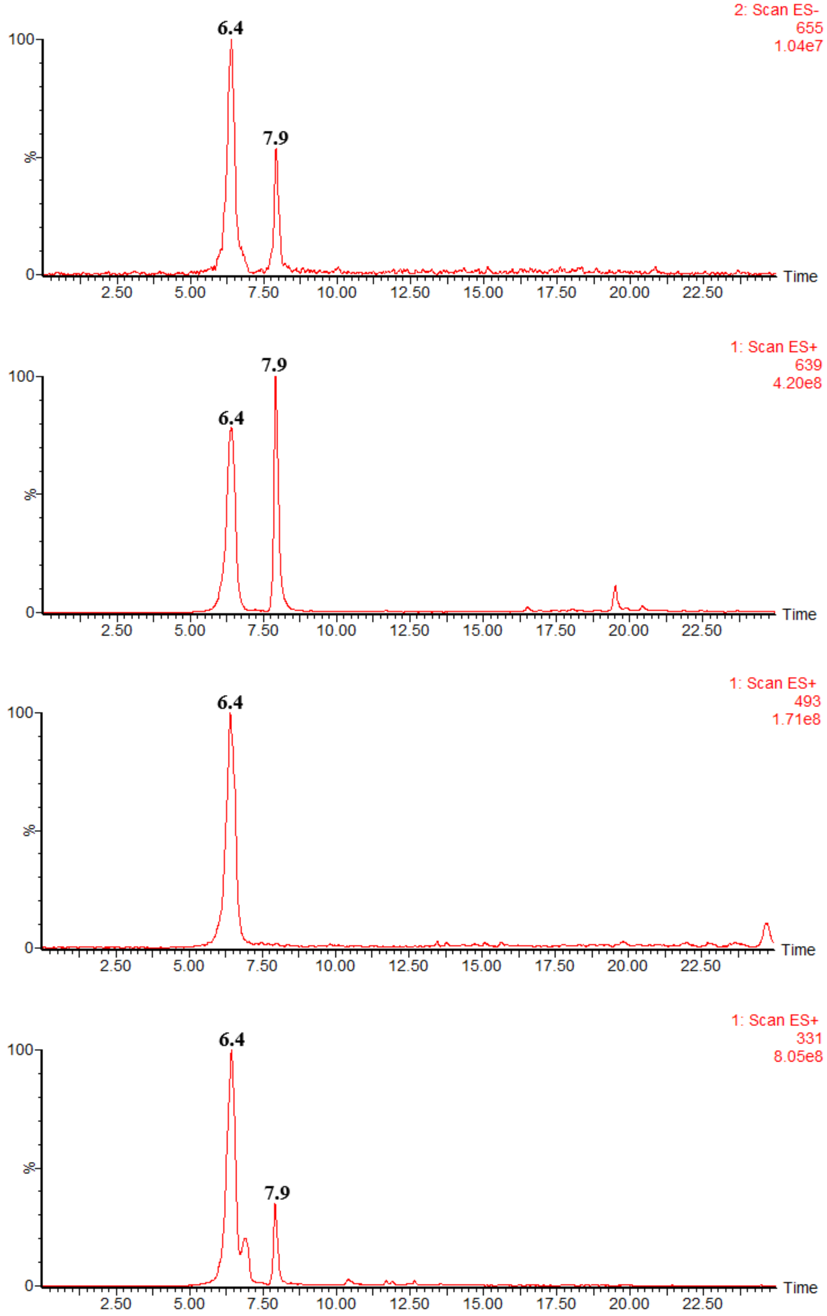

Figure S3. Single ion chromatograms obtained for malvidin 3-*O*-rutinoside (**4**, rt = 6.4 min) and malvidin 3-*O*-(6″-[*E*]-*p*-coumaroyl)glucoside (**6**, rt = 7.9 min), $[M{-}2H{+}H_2O]^-$ *m/z* 655, $[M]^+$ *m/z* 639, Y_1^+ *m/z* 493, Y_0^+ *m/z* 331.

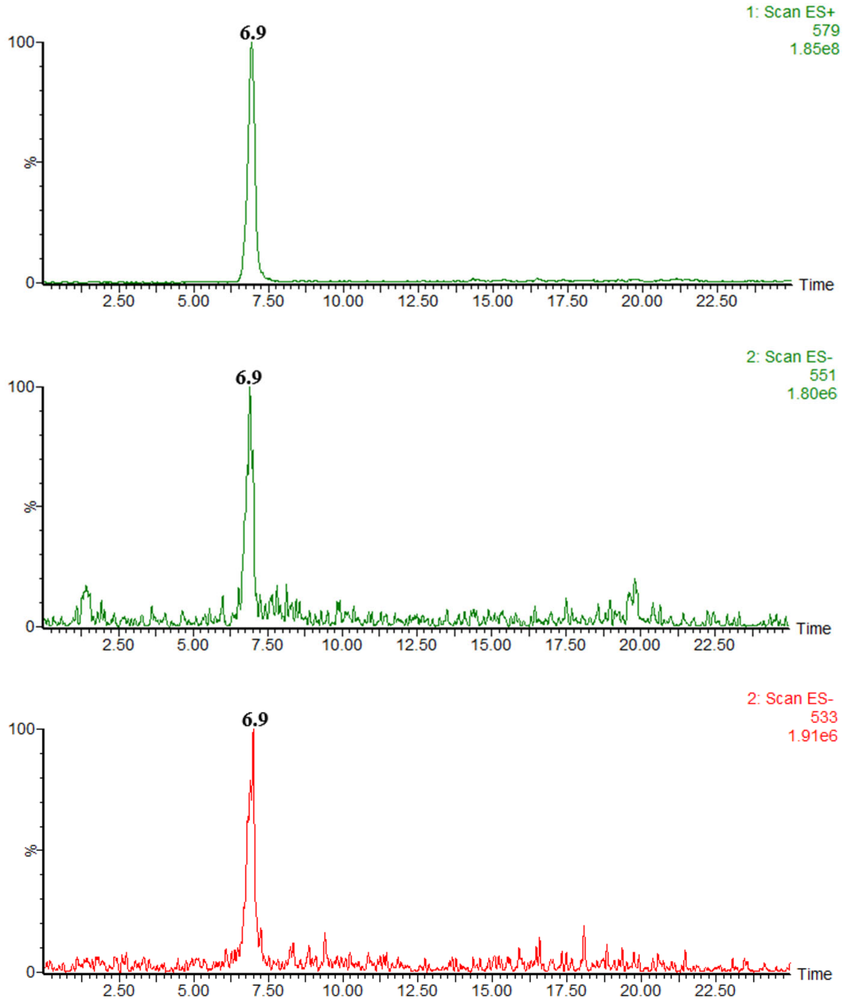

Figure S4. Exemplary single ion chromatograms obtained for malvidin 3-*O*-(6″-malonyl)glucoside (**7**), $[M]^+$ *m/z* 579, $[M{-}2H{+}H_2O{-}CO_2]^-$ *m/z* 551, $[M{-}2H{-}CO_2]^-$ *m/z* 533.

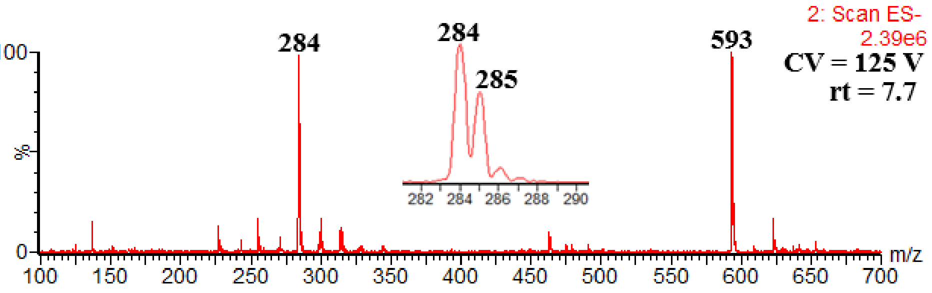

Figure S5. The ESI(−) mass spectrum of kaempferol 3-*O*-rutinoside (**9**).

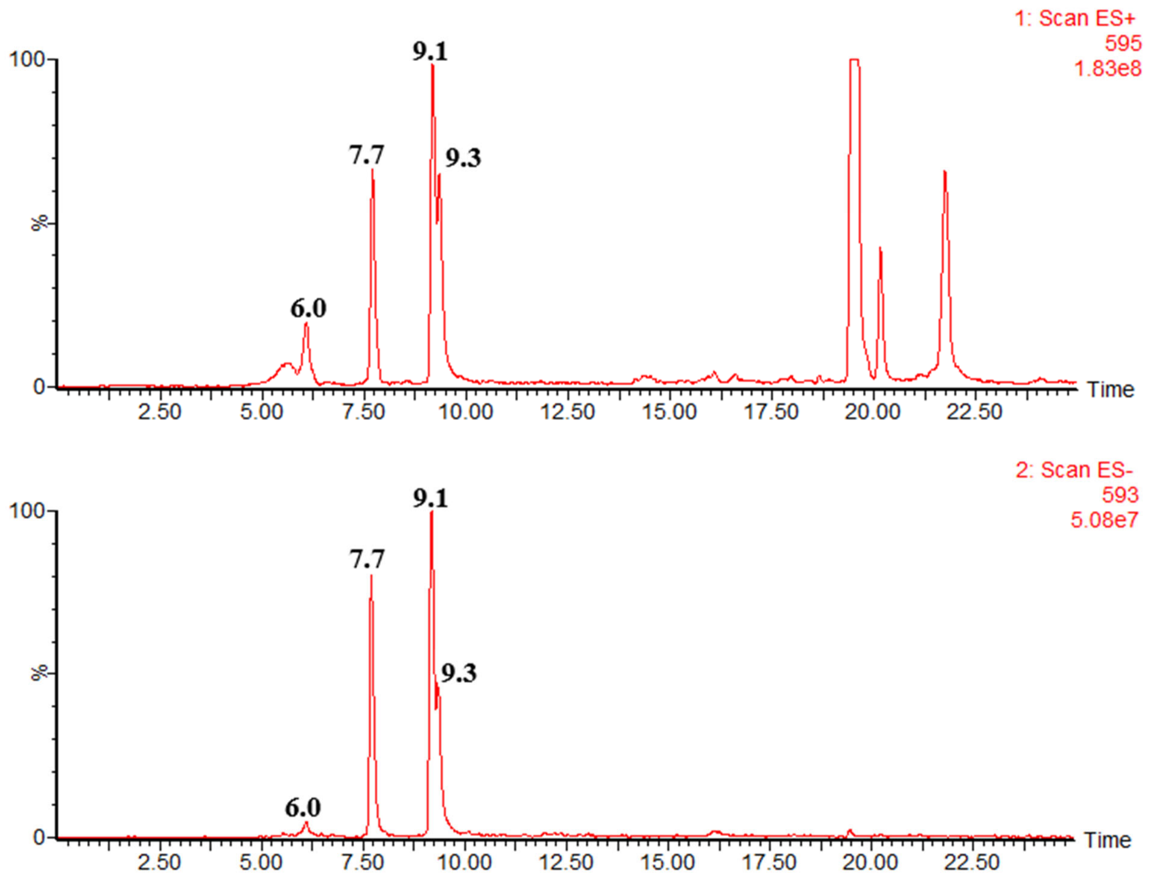

Figure S6. Single ion chromatograms obtained in the positive (*m/z* 595) and negative ion mode (*m/z* 593) for cyanidin 3-*O*-rutinoside (**1**, 6.0 min), kaempferol 3-*O*-rutinoside (**9**, 7.7 min), kaempferol 3-O-(6″-[E]-*p*-coumaroyl)glucoside (isomer *trans*, **15a**, 9.1 min) and kaempferol 3-O-(6″-[Z]-*p*-coumaroyl)glucoside (isomer *cis*, **15b**, 9.3 min).

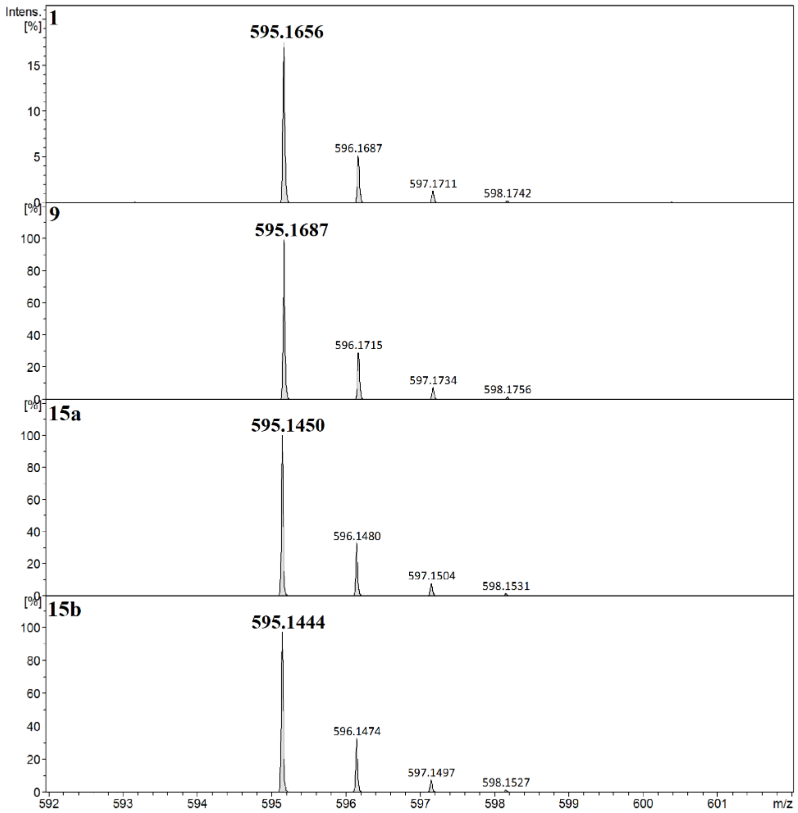

Figure S7. The high resolution mass spectra of **1**, **9**, **15a** and **15b**.

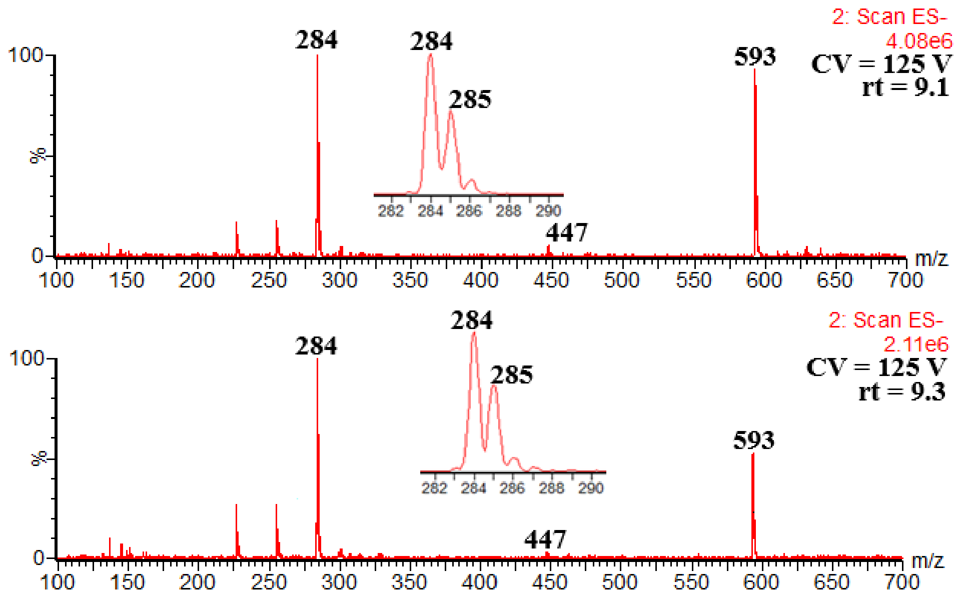

Figure S8. The ESI(−) mass spectra of isomers **15a** and **15b** (rt = 9.1 and rt = 9.3, respectively) obtained in the negative ion mode.

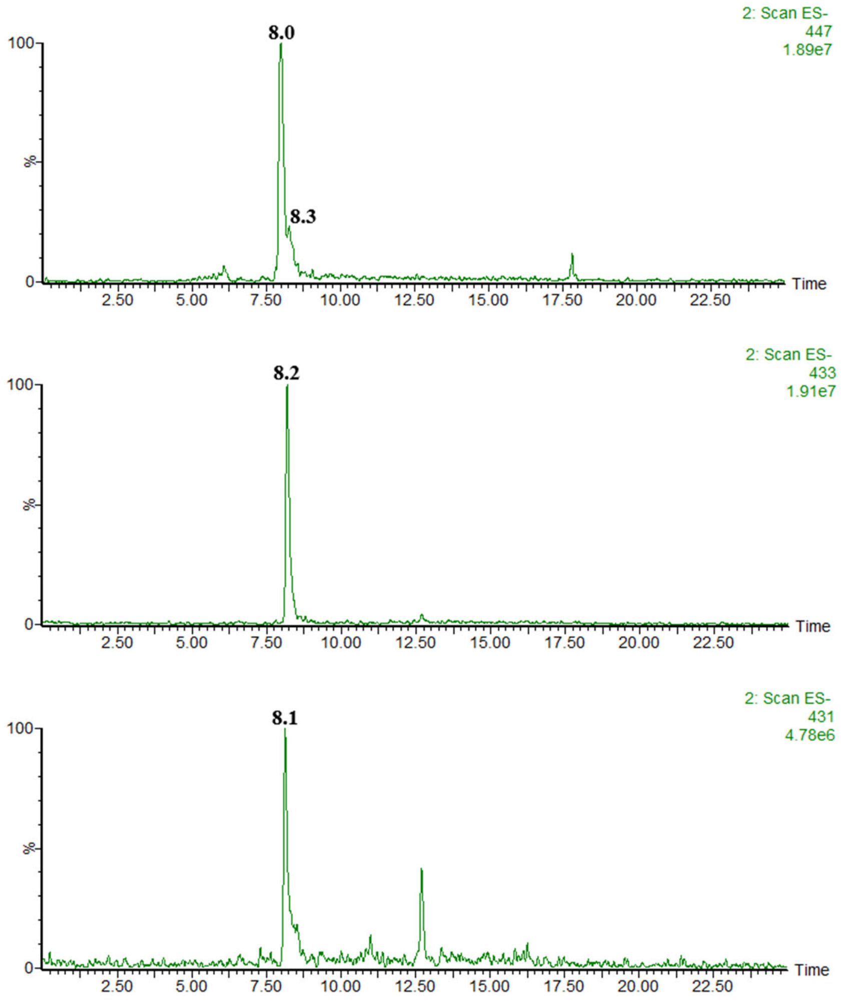

Figure S9. Single ion chromatograms obtained for [M-H]⁻ ions of apigenin 7-*O*-glucoside (**16**, *m/z* 431, rt = 8.1 min), luteolin 4'-*O*-glucoside (**17**, *m/z* 447, rt = 8.3 min), kaempferol 3-*O*-glucoside (**10**, *m/z* 447, rt = 8.0 min), and naringenin 7-*O*-glucoside (**18**, *m/z* 433, rt = 8.2 min).
